# Prevalence of interstitial pneumonia suggestive of COVID-19 at ^18^F-FDG PET/CT in oncological asymptomatic patients in a high prevalence country during pandemic period: a national multi-centric retrospective study

**DOI:** 10.1007/s00259-021-05219-0

**Published:** 2021-02-09

**Authors:** Domenico Albano, Francesco Bertagna, Pierpaolo Alongi, Sergio Baldari, Alfonso Baldoncini, Mirco Bartolomei, Franco Boccaletto, Michele Boero, Eugenio Borsatti, Andrea Bruno, Luca Burroni, Francesca Capoccetti, Massimo Castellani, Anna Rita Cervino, Franca Chierichetti, Andrea Ciarmiello, Angelo Corso, Alberto Cuocolo, Maria Luisa De Rimini, Desiree’ Deandreis, Massimo Eugenio Dottorini, Filomena Esposito, Mohsen Farsad, Massimo Gasparini, Chiara Maria Grana, Michele Gregianin, Luca Guerra, Fabio Loreti, Andrea Lupi, Gianluigi Martino, Elisa Milan, Sergio Modoni, Silvia Morbelli, Alfredo Muni, Emanuele Nicolai, Barbara Palumbo, Sergio Papa, Alberto Papaleo, Riccardo Pellerito, Carlo Poti, Pasquale Romano, Claudio Rossetti, Pierluigi Rossini, Giuseppe Rubini, Livia Ruffini, Gianmauro Sacchetti, Giordano Savelli, Saverio Schiavariello, Roberto Sciagrà, Rosa Sciuto, Ettore Seregni, Stelvio Sestini, Michele Sicolo, Angela Spanu, Giovanni Storto, Massimo Tosti Balducci, Giuseppe Trifirò, Annibale Versari, Alberto Vignati, Duccio Volterrani, Maria Lucia Calcagni, Maria Cristina Marzola, Antonio Garufo, Laura Evangelista, Roberto Maroldi, Orazio Schillaci, Raffaele Giubbini, On the behalf of Italian Association of Nuclear Medicine (AIMN), M. Bonacina, R. Laudicella, M. Spallino, A. Palucci, M. Cuzzocrea, M. Donner, S. Maurea, F. Ceci, F. Dei Rossi, B. Tranfaglia, A. Sartorello, P. Gandolfo, A. Buschiazzo, A. Scarale, M. Kirienko, N. Merenda, A. G. Nappi, R. Durmo, C. Vellani, V. Ceriani

**Affiliations:** 1 Nuclear Medicine, University of Brescia and ASST Spedali Civili Brescia, Brescia, Italy; 2 Unit of Nuclear Medicine, Fondazione Istituto G.Giglio, 90015 Cefalù, Italy; 3 Department of Biomedical and Dental Sciences and of Morpho-Functional Imaging, Nuclear Medicine Unit, University of Messina, 98125 Messina, Italy; 4 Nuclear Medicine Department, Ospedale San Donato, Arezzo, Italy; 5 Nuclear Medicine Department, Azienda Ospedaliero-Universitaria di Ferrara, Ferrara, Italy; 6 Nuclear Medicine Department, Treviso Hospital, Treviso, Italy; 7 Nuclear Medicine Unit, ARNAS G. Brotzu, Cagliari, Italy; 8 Nuclear Medicine, IRCCS CRO, Aviano, Italy; 9 Department of Nuclear Medicine, ASST Papa Giovanni XXIII, Bergamo, Italy; 10 Department of Nuclear Medicine, “Ospedali Riuniti di Torrette” Hospital, Ancona, Italy; 11 Service Department Macerata Hospital, ASUR Marche AV3, Nuclear Medicine Unit, Macerata, Italy; 12 Nuclear Medicine Department, Fondazione IRCCS Cà Grande Ospedale Maggiore Policlinico, Milan, Italy; 13 Nuclear Medicine Unit, Veneto Institute of Oncology IOV-IRCCS, Padua, Italy; 14 Nuclear Medicine, S. Chiara Hospital, Trento, Italy; 15 Nuclear Medicine Unit, S. Andrea Hospital, La Spezia, Italy; 16 Department of Nuclear Medicine, Sant’Anna Hospital, Como, Italy; 17 Department of Advanced Biomedical Sciences, University of Naples Federico II, Via Pansini 5, 80131 Naples, Italy; 18 Nuclear Medicine Unit, AO Ospedali Dei Colli, Monaldi Hospital, Naples, Italy; 19 Division of Nuclear Medicine, Department of Medical Sciences, University of Turin, AOU Città della Salute e della Scienza, 10126 Turin, Italy; 20 Department of Diagnostic Imaging, Nuclear Medicine Unit, Ospedale “S. Maria della Misericordia”, Perugia, Italy; 21 Nuclear Medicine, Neuromed, Pozzilli, Italy; 22 Nuclear Medicine, Central Hospital Bolzano, Bolzano, Italy; 23 Department of Nuclear Medicine, IRCCS MultiMedica Sesto San Giovanni, Milan, Italy; 24 Division of Nuclear Medicine, European Institute of Oncology, IRCCS, Milan, Italy; 25 Nuclear Medicine Unit, Veneto Institute of Oncology IOV-IRCCS, Castelfranco Veneto, Italy; 26 Nuclear Medicine, University of Milan Bicocca and ASST Ospedale San Gerardo, Monza, Italy; 27 Nuclear Medicine Unit, S. Maria Hospital, Terni, Italy; 28 Division of Nuclear Medicine, Ospedale S. Bortolo, Vicenza, Italy; 29 Unit of Nuclear Medicine, Department of Radiology and Clinical Radiotherapy, SS Annunziata Hospital, Chieti, Italy; 30 Nuclear Medicine Department, University Hospital Ospedali Riuniti, Foggia, Italy; 31 IRCCS Ospedale Policlinico San Martino, Genoa, Italy; 32 Department of Health Science (DISSAL), University of Genoa, Genoa, Italy; 33 Nuclear Medicine Department, Azienda Ospedaliera S.S. Antonio e Biagio e Cesare Arrigo Hospital, Alessandria, Italy; 34 IRCCS SDN, Naples, Italy; 35 Section of Nuclear Medicine and Health Physics, Department of Medicine and Surgery-University of Perugia, Perugia, Italy; 36 Unit of Diagnostic Imaging and Stereotactic Radiosurgery, CDI Centro Diagnostico Italiano,, Via Saint Bon 20, 20147 Milan, Italy; 37 Nuclear Medicine Department, S. Croce e Carle Hospital Cuneo, Cuneo, Italy; 38 Department of Nuclear Medicine, Mauriziano Hospital, Turin, Italy; 39 Unit of Nuclear Medicine, Aosta Regional Hospital, Aosta, Italy; 40 Department of Nuclear Medicine, Studio Radiologico Guidonia, Guidonia, Italy; 41 Nuclear Medicine, ASST Grande Ospedale Metropolitano Niguarda, Milan, Italy; 42 Nuclear Medicine, Azienda Socio Sanitaria Territoriale di Mantova. Ospedale C. Poma, Mantova, Italy; 43 Nuclear Medicine Unit, Department of Interdisciplinary Medicine, AOU Policlinico, University of Bari, Bari, Italy; 44 Nuclear Medicine Unit, Azienda Ospedaliero-Universitaria di Parma, Parma, Italy; 45 Nuclear Medicine Department, AOU Maggiore della Carità, Novara, Italy; 46 Nuclear Medicine Department, Fondazione Poliambulanza, Brescia, Italy; 47 Nuclear Medicine Unit, ASM Matera, Matera, Italy; 48 Nuclear Medicine Unit, Department of Experimental and Clinical Biomedical Sciences “Mario Serio”, University of Florence, Florence, Italy; 49 Nuclear Medicine Unit, IRCCS-Regina Elena National Cancer Institute, 00144 Rome, Italy; 50 Division of Nuclear Medicine, Fondazione IRCCS Istituto Nazionale dei Tumori, Milan, Italy; 51 Unit of Nuclear Medicine, Department of Diagnostic Imaging, N.O.P. - S. Stefano, U.S.L. Toscana Centro, Prato, Italy; 52 Nuclear Medicine Unit, Dell’Angelo Hospital, Mestre-Venezia, Italy; 53 Nuclear Medicine Unit, Department of Medical, Surgical and Experimental sciences, University of Sassari, Sassari, Italy; 54 Nuclear Medicine Department, IRCCS CROB, Referral Cancer Center of Basilicata, 85028 Rionero in Vulture, Italy; 55 Nuclear Medicine Department, Azienda Usl Toscana sud est, Arezzo, Italy; 56 Nuclear Medicine Department, ICS Maugeri SpA SB-IRCCS, Pavia, Italy; 57 Nuclear Medicine Unit, Azienda AUSL-IRCCS di Reggio Emilia, Reggio Emilia, Italy; 58 Nuclear Medicine Department, ASST Ovest Milanese, Legnano, Italy; 59 Regional Center of Nuclear Medicine, University Hospital of Pisa, Pisa, Italy; 60 Istituto di Medicina Nucleare, Università Cattolica del Sacro Cuore, & UOC di Medicina Nucleare, Dipartimento di Diagnostica per Immagini, Radioterapia Oncologica ed Ematologia, Fondazione Policlinico Universitario “A. Gemelli” IRCCS, L.go Agostino Gemelli 8, 00168 Rome, Italy; 61 Department of Nuclear Medicine PET/CT Centre, S. Maria della Misericordia Hospital, 45100 Rovigo, Italy; 62 Nuclear Medicine Department, ASP Agrigento, Agrigento, Italy; 63 Nuclear Medicine Unit, Department of Medicine-DIMED, University of Padova, 35128 Padova, Italy; 64 Department of Radiology, University of Brescia, ASST Spedali Civili Brescia, Brescia, Italy; 65 Department of Biomedicine and Prevention, Tor Vergata University Hospital, Rome, Italy

**Keywords:** ^18^F-FDG PET/CT, COVID-19, Interstitial pneumonia, SARS-CoV-2, Asymptomatic, Incidental findings

## Abstract

**Purpose:**

To assess the presence and pattern of incidental interstitial lung alterations suspicious of COVID-19 on fluorine-18-fluorodeoxyglucose positron emission tomography (PET)/computed tomography (CT) ([^18^F]FDG PET/CT) in asymptomatic oncological patients during the period of active COVID-19 in a country with high prevalence of the virus.

**Methods:**

This is a multi-center retrospective observational study involving 59 Italian centers. We retrospectively reviewed the prevalence of interstitial pneumonia detected during the COVID period (between March 16 and 27, 2020) and compared to a pre-COVID period (January–February 2020) and a control time (in 2019). The diagnosis of interstitial pneumonia was done considering lung alterations of CT of PET.

**Results:**

Overall, [^18^F]FDG PET/CT was performed on 4008 patients in the COVID period, 19,267 in the pre-COVID period, and 5513 in the control period. The rate of interstitial pneumonia suspicious for COVID-19 was significantly higher during the COVID period (7.1%) compared with that found in the pre-COVID (5.35%) and control periods (5.15%) (*p* < 0.001). Instead, no significant difference among pre-COVID and control periods was present. The prevalence of interstitial pneumonia detected at PET/CT was directly associated with geographic virus diffusion, with the higher rate in Northern Italy. Among 284 interstitial pneumonia detected during COVID period, 169 (59%) were FDG-avid (average SUVmax of 4.1).

**Conclusions:**

A significant increase of interstitial pneumonia incidentally detected with [^18^F]FDG PET/CT has been demonstrated during the COVID-19 pandemic. A majority of interstitial pneumonia were FDG-avid. Our results underlined the importance of paying attention to incidental CT findings of pneumonia detected at PET/CT, and these reports might help to recognize early COVID-19 cases guiding the subsequent management.

**Supplementary Information:**

The online version contains supplementary material available at 10.1007/s00259-021-05219-0.

## Introduction

An aggressive acute respiratory disease caused by a novel coronavirus of zoonotic origin, called COVID-19, occurred during December 2019 in Wuhan, China, and then spread everywhere becoming a pandemic. Europe, and especially Northern Italy, was hit by this infection with the maximum incidence during March [1, 2]. COVID-19 may present with no specific signs and symptoms like fever, dyspnea, and cough, but in most cases, the patients may be also asymptomatic. This scenario is the most dangerous because they can be potential sources of infection for a whole population. The exact incidence of asymptomatic patients remains unclear and yet under debate. Furthermore, it is well known that often clinical symptoms appear when the infection is in the peak phase.

Until now, reverse transcriptase-polymerase chain reaction (RT-PCR) is considered to be the gold standard for the diagnosis of COVID-19 infection, but a high false-negative rate is reported, which can cause a miss or delay in the effective diagnosis and increase the risk of spread of the epidemic [3]. The reasons of these false negative reports are several: the lack of shared and standard operation procedures and validation across different laboratories and hospitals, different disease stages of infection at time of examination, different viral loads, and the potential mutation rate of the virus.

Published data [4–6] about the potential role of radiological tools, such as chest X-rays and computed tomography (CT), in detecting COVID-19-related interstitial pneumonia are available, showing good accuracy and describing as typical pattern of presentation the presence of ground-glass opacities (GGOs) or bilateral pulmonary consolidations in multiple lobular and sub-segmental areas [2, 7]. For this reason, some authors suggest considering the combination of clinical, imaging, and laboratory reports to make the final diagnosis, and in some cases, the CT morphological pattern seems to be better than the RT-PCR test [8, 9].

Also fluorine-18-fluorodeoxyglucose positron emission tomography/CT ([^18^F]FDG PET/CT) is able to reveal lung alterations suspicious for COVID-19 pneumonia despite the sub-optimal diagnostic power of CT (generally low dose) and several cases of incidental interstitial pneumonia COVID-19-related detected by [^18^F]FDG PET/CT in asymptomatic patients are present in literature [10–19], but these reports are usually case reports or single-center experience.

The aim of this study was to compare the prevalence of incidental interstitial pneumonia suspected for COVID-19 in three different periods (“COVID,” “pre-COVID,” and “control” periods) in a country with high prevalence of infection and to describe the main radiological and metabolic features of this pneumonia during the pandemic time.

## Materials and methods

### Study design

This is a multi-center retrospective observational study which saw the participation of 59 centers from all over Italy.

This study was approved by ethics committee (NP 4049) of ASST *Spedali Civili* of Brescia.

### Population

**Fig. 1:**
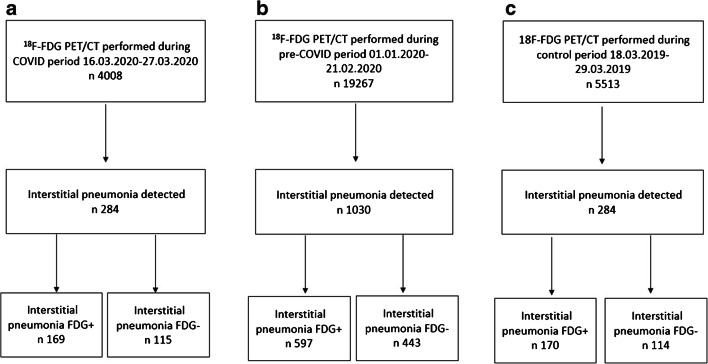
Flowchart of the cohort selection in the COVID period (**a**), pre-COVID period (**b**), and control period (**c**)

We retrospectively reviewed the [^18^F]FDG PET/CT scans performed in asymptomatic patients for routine oncological purpose in our centers in three different periods: (a) from March 16, 2020, to March 27, 2020 (*n* = 4008), called conventionally “COVID-19 period” being considered a pandemic time; (b) from January 1, 2020, to February 21, 2020 (*n* = 19,267), called conventionally “pre-COVID-19 period” considered a time immediately before the virus spread in our country; (c) from March 18, 2019, to March 29, 2019 (*n* = 5513), called conventionally “control period” considered a COVID-free time being about 1 year before the pandemic (Fig. 1).

The period from March 9, 2020, was the beginning of the social containment in Italy with the approval of the national lockdown; thus, the patients were determined to be asymptomatic for COVID-19 infection verifying body temperature and excluding any signs or symptoms related to virus infection by a short anamnesis before accessing the nuclear medicine departments. No suspicion of viral infection was present in the days before or at the time of the PET/CT scan in any of our patients. The pre-COVID-19 period was included in our analysis to investigate if immediately before the official virus outbreak, COVID-19 was already diffused in the country, maybe in a subtle way.

Inclusion criteria: patients more than 18 years old at time of PET/CT scan, patients studied for oncological purposes (staging, restaging, or follow-up), patients without any signs or symptoms suspected for COVID-19 infection at time of PET/CT or on the days before

Exclusion criteria: patients younger than 18 years old and patients with concomitant known inflammatory lung diseases

When possible, patients with incidental interstitial pneumonia detected by PET/CT underwent a subsequent nasopharyngeal swab by RT-PCR test to confirm the diagnosis of COVID-19 infection. RT-PCR was performed the same day or after the scan within 3 days.

We reviewed the medical and pathology reports of the patients with incidental interstitial pneumonia discovered during the “COVID period”: epidemiological (age, gender), clinical (oncological disease, clinical indication of PET/CT), and biochemical (lymphocyte count, erythrocyte sedimentation rate (ESR) level, C-reactive protein (CRP) level) features, morphological/radiological pattern by chest CT of PET and metabolic features by [^18^F]FDG PET/CT were collected and analyzed in all patients.

### ^18^F-FDG PET/CT imaging and interpretation

All [^18^F]FDG PET/CT scans were performed following the international guidelines of the European Association of Nuclear Medicine [20]. The PET/CT scans were performed on 69 scanners at the 59 participating centers. The patients underwent [^18^F]FDG PET/CT after at least 6 h of fasting and with glucose level lower than 150 mg/dl.

The injected activity of [^18^F]FDG was dependent on the PET scanner used and the patient weight according to local protocols. Whole-body imaging began at 60 ± 10 min after radiotracer injection and was acquired from the skull base to the mid-thigh and reconstructed using attenuation correction.

The PET/CT images were reviewed by local expert nuclear medicine physicians and if necessary by local experienced thoracic radiologists. The diagnosis of highly suspicious viral interstitial pneumonia was based on the findings of pulmonary infiltrates, GGOs, and subpleural pseudo-nodular mixed ground-glass and consolidation areas reviewing chest CT of PET. CT pattern of lung alterations was considered to define the presence of pneumonia. Laterality, localization, morphological pattern (ground-grass capacities, consolidation areas, nodules), distribution (peripheral or central, or both), presence of enlarged thoracic lymph nodes, and pleural effusion were described for each scan.

Interstitial lung abnormalities were subsequently evaluated both visually and semiquantitatively by FDG PET/CT.

Every focal tracer uptake deviating from physiological distribution and with a maximum standardized uptake value (SUVmax) higher than 2.5 was regarded as positive for FDG-avidity.

Maximum and mean standardized uptake values (SUVmax and SUVmean) of the lung abnormalities and mediastinal lymph nodes were semi-automatically calculated using local workstation. We measured the SUV of detectable lesions by drawing a region of interest (ROI) over the area of maximum activity. Furthermore, SUVmax of mediastinum was calculated at the aortic arch by use of axial PET images with a round-shaped 10-mm ROI not involving the wall of the vessel; SUVmax of the liver was calculated at the VIII hepatic segment of axial PET images using the same ROI. The ratios between SUVmax of hypermetabolic lung alterations and SUVmax of mediastinum and liver were calculated for each patient.

### Statistical analysis

Statistical analyses were performed using MedCalc Software version 18.1. The descriptive analysis of categorical variables is characterized by the calculation of simple and relative frequencies, while the numeric variables by median, mean, minimum, and maximum values.

Chi-square (*χ*^2^) test and Student’s *t* test were performed to compare the distributions of categorical and continuous variables, respectively. To compare the proportions of interstitial pneumonia observed in the three periods, the McNemar’s test was used. A *p* value < 0.05 was considered to indicate statistical significance.

## Results

### National analysis

Overall, [^18^F]FDG-PET/CT was performed on 4008 patients in the COVID period, 19,267 in the pre-COVID period, and 5513 in the control period.

**Fig. 2:**
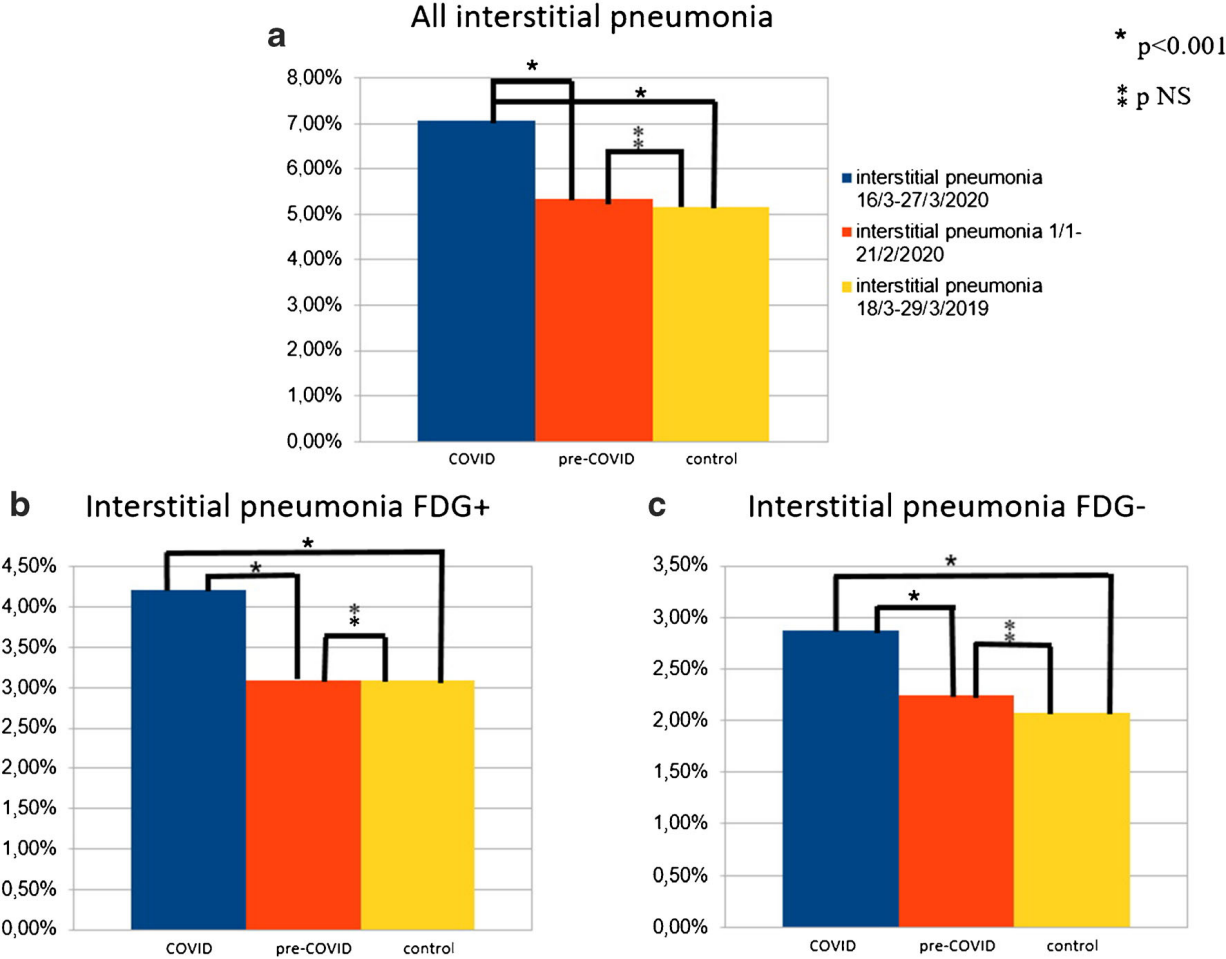
Histogram graphs representing all interstitial pneumonia (**a**), interstitial pneumonia [^18^F]FDG positive (**b**), and interstitial pneumonia [^18^F]FDG negative (**c**) detected in the three periods in the country

In the COVID period, 284/4008 (7.1%) [^18^F]FDG PET/CT scans demonstrated the presence of lung abnormalities consistent of interstitial pneumonia, while 1030/19,267 (5.35%) in the pre-COVID period and 284/5513 (5.15) in the control period (Fig. 1; Table 1). The rate of interstitial pneumonia was significantly higher in the COVID period than that in the pre-COVID and control time (*p* < 0.001 and *p* < 0.001, respectively). Instead, no significant difference among the pre-COVID and control periods was present (*p* = 0.569) (Fig. 2a). Similar evidences were derived also considering the prevalence of [^18^F]FDG-avid interstitial pneumonia during the COVID period (4.2% of all examinations) in comparison with pre-COVID (3.1% of all examinations; *p* < 0.001) and control (3.1% of all examinations; *p* < 0.001) periods (Fig. 2b) and the prevalence of [^18^F]FDG-negative interstitial pneumonia during the COVID period (2.9% of all examinations) in comparison with pre-COVID (2.25% of all examinations; *p =* 0.018) and control (2.1% of all examinations; *p* = 0.011) periods (Table 1; Fig. 2c).

**Table 1:** Prevalence of incidental interstitial pneumonia in the three periods analyzed

	Number of PET/CT scans	Number of interstitial pneumonia	Number of interstitial pneumonia FDG+	Number of interstitial pneumonia FDG−
National data (59 centers)				
COVID period	4008	284 (7.1%)	169 (4.2%)	115 (2.9%)
Pre-COVID period	19,267	1030 (5.35%)	597 (3.1%)	433 (2.25%)
Control period	5513	284 (5.15%)	170 (3.1%)	114 (2.1%)
Northern Italy data (35 centers)				
COVID period	2493	196 (7.9%)	117 (4.7%)	79 (3.2%)
Pre-COVID period	12,716	721 (5.7%)	433 (3.4%)	288 (2.3%)
Control period	3428	191 (5.6%)	112 (3.3%)	79 (2.3%)
Central Italy data (12 centers)				
COVID period	674	26 (3.9%)	19 (2.8%)	7 (1.1%)
Pre-COVID period	3115	91 (2.9%)	63 (2%)	28 (0.9%)
Control period	800	27 (3.4%)	20 (2.5%)	7 (0.9%)
Southern Italy–Islands data (12 centers)				
COVID period	841	62 (7.4%)	33 (3.9%)	29 (3.5%)
Pre-COVID period	3436	278 (6.3%)	101 (2.9%)	117 (3.4%)
Control period	1285	66 (5.1%)	38 (2.9%)	28 (2.2%)

Among 284 interstitial pneumonia detected during COVID period, 169 (59%) were FDG-avid showing an increased [^18^F]FDG uptake corresponding to the lung abnormalities, while the remaining 115 (41%) had no significant [^18^F]FDG uptake. Of 1030 interstitial pneumonia discovered during the pre-COVID time, 597/1030 (58%) were FDG positive, and the remaining 433 (42%) were negative. In the control period, the interstitial pneumonia [^18^F]FDG positive were 170 (59%), and the interstitial pneumonia [^18^F]FDG negative were 114 (41%). The prevalence of [^18^F]FDG-avidity among all interstitial pneumonia was not significantly different comparing the three periods.

### Macro-area analysis

We conventionally performed a subanalysis dividing all national data into three geographic macro-areas: Northern Italy, Central Italy, and Southern Italy and Islands (Supplemental Fig. 1). Two-thousand four hundred ninety-three [^18^F]FDG PET/CT scans were performed in Northern Italy (35 centers) during the COVID period; in the same period, 674 [^18^F]FDG PET/CT scans were acquired in Central Italy (12 centers) and 841 in Southern Italy and Islands (12 centers).

In the COVID period, the prevalences of interstitial pneumonia were 7.9% in the Northern Italy, 3.9% in the Central Italy, and 7.4% in the Southern Italy; in the pre-COVID period were 5.7%, 2.9%, and 6.3%, respectively; in the control time 5.6%, 3.4%, and 5.1%, respectively (Table 1).

**Fig. 3:**
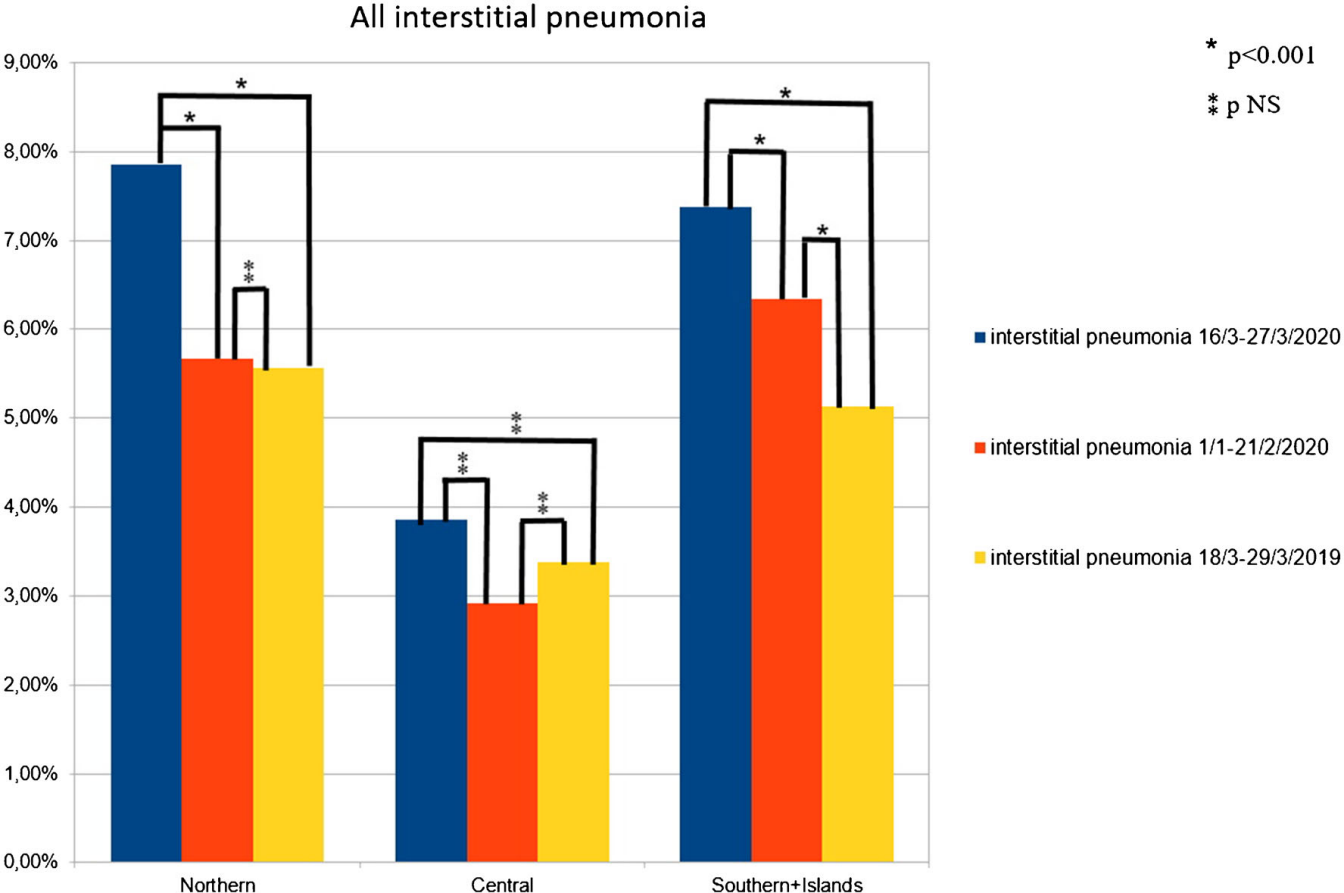
Histogram graphs representing all interstitial pneumonia detected in the three periods in the Northern, Central, and Southern Italy

In Northern Italy, the prevalence of interstitial pneumonia was significantly higher during the COVID period than the other two time periods (*p* < 0.001 for both), while no difference in detection rate between pre-COVID and control periods (*p* = 0.825) was registered. For Southern Italy–Islands, similar evidences were obtained (COVID vs pre-COVID *p* < 0.001; COVID vs control *p* < 0.001; pre-COVID vs control *p* = 0.005) (Fig. 3).

Instead, considering Central Italy, no significant differences among interstitial pneumonia detected in the three times were present (COVID vs pre-COVID *p* = 0.202; COVID vs control *p* = 0.503; pre-COVID vs control *p* = 0.620) (Fig. 3).

No significant differences considering median age of patients with interstitial pneumonia in the three macro-areas were discovered (median age in Northern Italy 68 years, 66 years in Central Italy, and 65 years in Southern Italy–Islands).

### Features of patients with interstitial pneumonia at the COVID period

he mean age of patients with incidental interstitial pneumonia detected during COVID-period was 66.3 years (range 18–90), and there was a prevalence of male gender (*n* = 159, 56%).

The majority of imaging studies with incidental interstitial pneumonia suspected for COVID-19 was performed for diagnosis/staging (*n* = 144) and restaging/treatment response evaluation (*n* = 144); the remaining 34 scans were acquired for follow-up purpose. The most frequent oncological disease studied was lung carcinoma (*n* = 69, 24%), followed by lymphoma (*n* = 60, 21%), and breast (*n* = 36, 13%) and head and neck (*n* = 28, 10%) cancers. The majority of patients had higher ESR and/or CRP levels, consistent of active inflammation disease. The main demographic features are described in Table 2.

**Table 2:** The main clinical and demographic features of patients with interstitial pneumonia detected during “COVID” period

	*n* (%)
Age average ± SD (range)	66.3 ± 13.8 (18–90)
Gender F:M	89:159 (44%:56%)
Oncological disease	
Lymphoma	60 (21%)
Multiple myeloma	5 (2%)
Lung	69 (24%)
Breast	36 (13%)
Gastrointestinal	18 (6%)
Head and neck	28 (10%)
Melanoma	4 (2%)
Genitourinary	22 (8%)
Other primary	42 (14%)
Indication	
Staging/diagnosis	125 (44%)
Restaging/treatment response evaluation	126 (44%)
Follow-up	34 (12%)
Low lymphocyte count	38/118 (32%)
Increased ESR level	25/47 (53%)
Increased CPR level	68/94 (72%)

*F*, female; *M*, male; *ESR*, erythrocyte sedimentation rate; *CRP*, C-reactive protein

Abnormal CT findings were observed during the pandemic in 284 patients; in particular, CT abnormalities consisting of GGOs were registered in 148 cases (52%) and GGOs associated with consolidation areas in 117 cases (41%). In the remaining 19 cases (7%), only consolidation areas (5%) or other rare signs, like nodules (2%), were reported (Table 3**)**.

**Table 3:** Summary of the main radiological and metabolic features of patients with interstitial pneumonia detected during “COVID” period (*n* = 284)

	*n* (%)	Mean (range)
CT features		
Laterality		
Monolateral lung abnormalities	139 (49%)	
Right side	71	
Left side	68	
Bilateral lung abnormalities	145 (51%)	
Number of lobes involved		
1	32 (11%)	
2	67 (24%)	
> 2	185 (65%)	
Localization		
Right lung	216	
RUL:RML:RLL	158:100:198	
Left lung	213	
LUL:Li:LLL	146:99:208	
Alterations pattern		
Only GGO	148 (52%)	
Only consolidation areas	14 (5%)	
GGO + consolidation areas	117 (41%)	
Other	5 (2%)	
Distribution of lesions in lung		
Peripheral	200 (70%)	
Central	19 (7%)	
Peripheral + central	65 (23%)	
Bronchial wall thickening	68 (24%)	
Crazy paving pattern	118 (42%)	
Air bronchogram sign	109 (38%)	
Pleural effusion	44 (15%)	
Enlarged mediastinal lymph nodes	169 (59.5%)	
PET features		
FDG-positive lung alterations	169 (59%)	
SUVmax lung alterations		4.1 (0.9–14.6)
SUVmean lung alterations		2.6 (0.7–10)
Lesion to liver SUVmax ratio lung alterations		1.4 (0.3–4.7)
Lesion to blood pool SUVmax ratio lung alterations		1.9 (0.3–6.8)
FDG-positive mediastinal lymph nodes	106 (37%)	

*CT*, computed tomography; *PET*, positron emission tomography; *RUL*, right upper lobe; *RML*, right medium lobe; *RLL*, right lower lobe; *LUL*, left upper lobe; *Li*, lingula; *LLL*, left lower lobe; *GGO*, ground-glass opacity; *SUV*, standardized uptake value

Bronchial wall thickening sign was present in 24% and pleural effusion in 15%. In most patients, enlarged mediastinal lymph nodes were noted. In most cases (65%), more than two pulmonary lobes were involved, while there was no laterality prevalence (unilateral pneumonia in 139 patients and bilateral pneumonia in 145 patients). Among unilateral pneumonia, there was no prevalence for the right or left lung.

Most of interstitial pneumonia (59%) were FDG-avid showing an increased radiotracer uptake, defined as tracer uptake higher from physiological distribution and with a SUVmax higher than 2.5. In all population (interstitial pneumonia FDG-avid and not avid), the intensity of [^18^F]FDG uptake, in terms of SUVmax, ranged between 0.9 and 14.6 (average 4.1), and in terms of SUVmean ranged between 0.7 and 10 (average 2.6). Average lesion to liver SUVmax ratio and lesion to blood pool SUVmax ratio were 1.4 (range 0.3–4.7) and 1.9 (0.3–6.8), respectively. Only in 106 cases (37%) was the presence of mediastinal lymph nodes FDG-avid registered.

RT-PCR test was performed only in 46 patients (all from COVID period), being positive in 35 cases (76%) and negative in the remaining 11 cases (24%). Of 35 RT-PCR–positive patients, 27 had FDG-avid interstitial pneumonia and the remaining 8 FDG-negative, while among 11 negative RT-PCR patients, 8 had FDG-avid interstitial pneumonia and the remaining 3 negative.

## Discussion

It is well known that the lungs are the most frequent sites of incidental findings suspicious for COVID-19 at [^18^F]FDG PET/CT, and this evidence is directly related to the pulmonary tropism of this virus; interstitial pneumonia may be dangerous and lead to respiratory failure and also death if not treated successfully. Thus, an early detection of lung involvement from COVID-19 is mandatory and potentially useful to treat these patients effectively. This is even more crucial in asymptomatic or paucity symptomatic patients, which represent the majority of patients affected by COVID-19 and may be source of infection spread accidentally.

Our results suggest the idea that [^18^F]FDG PET/CT may be an indirect way to detect asymptomatic patients suspected for COVID-19 infection and lead them to subsequent analyses (such as diagnostic chest CT and/or RT-PCR test) to identify infectious patients.

Of course, the detection of interstitial pneumonia suspected for COVID-19 is done with the help of CT. Now, almost all PET scanners included CT as part of tomography. Thus, an accurate analysis of CT images seems to be mandatory also for nuclear medicine physicians with the aim of integrating morphological and metabolic data in the same moment. As reported before [16–19], we defined viral interstitial pneumonia suspected for COVID-19 infection if pulmonary infiltrates, GGOs, and subpleural pseudo-nodular mixed ground-glass and consolidation areas were present at CT of PET.

The typical CT signs of interstitial pneumonia suspicious for COVID-19 are ground-glass opacities and/or pulmonary consolidation areas as demonstrated by our data: in our population, only GGOs were present in 52% of cases, GGOs associated with consolidation areas in 41% of cases and only consolidation areas in 5%. These data are similar to previously published [21–24]. Theoretically, also rarer lung alterations are possible (2% in our analysis) like nodular areas. In our population, there was a similar pulmonary spread of lung alterations: in 51% of patients, both the lungs were involved, in contrast with 49% with unilateral disease. This evidence is partially in contrast with previous reports [21–24] where a bilateral disease was often reported. This discrepancy may be probably explained by the early phase of COVID-19 infection detected by our scans, performed in all asymptomatic patients, thus with no clinical evidence of COVID-19 disease at the time of PET/CT scan. However, in most cases (65%), more than two lobes were hit by lung lesions, and there was a prevalence for lower lobes.

Of course, radiological or metabolic features at PET/CT were not specific for COVID-19, and they might not be applied for the differential diagnosis of pulmonary infections or inflammatory diseases. High-resolution chest CT remains the most accurate imaging tool to study COVID-19 interstitial pneumonia; until now, there are no findings suggesting an added value of [^18^F]FDG PET/CT compared to chest CT in the management or outcome of patients with COVID-19 infection. Furthermore, [^18^F]FDG PET/CT is surely a more complex tool than chest CT leading to a possible increased risk of disease spreading due to the longer time of [^18^F]FDG PET/CT procedure. However, accidental detection of lung alterations suspected for COVID-19 may be recognized also with [^18^F]FDG PET/CT, and in these cases, [^18^F]FDG PET/CT could be considered an indirect way to suspect COVID-19 infection.

COVID-19-related interstitial pneumonia was [^18^F]FDG-avid in most cases with heterogeneous [^18^F]FDG uptake; in our analysis, we reported an FDG avidity of 59% with an average SUVmax of 4.1 (range 0.9–14.6) and an average SUVmean of 2.6 (range 0.7–10). Instead, mediastinal lymph node involvement at PET/CT was discovered in only 37% of all patients studied during the COVID period. These evidences underlined the aggressiveness of interstitial pneumonia which reflect the high metabolic activity at PET and were similar to other studies [16–19].

The findings of an increased FDG uptake in pulmonary or lymph nodal lesions in patients with COVID-19 infection are not unexpected as acute inflammatory conditions and infectious pulmonary alterations are usually characterized by increased ^18^F-FDG uptake [25]. The ability of [^18^F]FDG PET/CT to detect areas of infection and inflammation is mainly associated with the glycolytic activity of the cells involved in the inflammatory process. In this regard, it has already been demonstrated that cells involved in infection and inflammation are mainly neutrophils and monocyte/macrophages, which are able to express high levels of glucose transporters and hexokinase activity. It was demonstrated that interstitial pneumonia in COVID-19 patients was characterized by alveolar cell damage which is a consequence of a systemic hyperinflammation condition defined as macrophage activation syndrome or cytokine storm [26–28]; the alveolar macrophages produce pro-inflammatory cytokines and chemokines, thus resulting in a cytokine storm.

Our results showed a higher rate of interstitial pneumonia during the COVID period compared with the pre-COVID and control periods, in agreement with previous local results [16–19], but evaluating a larger population and representative of Italy. This national evidence was confirmed also analyzing macro-areas like Northern Italy and Southern Italy–Islands. On the other hand, when analyzing data from Central Italy, a higher rate of interstitial pneumonia during COVID time was reported but not statistically different compared with the other periods. These results may be explained by the low diffusion of the virus in Central Italy in the period analyzed and the small number of PET/CT scans performed in the same period in the centers recruited in Central Italy (lower than the Northern and Southern). These results confirmed the concept that the rates of incidental pneumonia findings were directly related to the prevalence of COVID-19 in different countries and regions. The highest prevalence of interstitial pneumonia was registered in Northern Italy, the region most affected in March 2020 by the infection.

Instead, when comparing pre-COVID and control periods, no significant differences in interstitial pneumonia detection were observed, underlying that probably the spread of COVID-19 in January and February 2020 in Italy was not so significant.

The analysis on the subgroups of patients with FDG-avid or not FDG-avid interstitial pneumonia confirmed the same evidences with a significant higher prevalence in the COVID period.

Our results stressed strongly that nuclear medicine physicians should pay attention to incidental CT findings of interstitial pneumonia (especially when morphological pattern is suspected for COVID-19 infection) detected with [^18^F]FDG PET/CT [29, 30], and these reports might help to recognize early COVID-19 cases guiding the subsequent management. Nuclear medicine physicians and staff should be on alert since incidental abnormal findings suspicious for COVID-19 may occur on [^18^F]FDG PET/CT scans, especially when a new epidemic peak is expected.

One of the limitations of this study was the lack of molecular confirmation (RT-PCR) of COVID-19 infection in all patients with interstitial pneumonia detected during the COVID period; this was due to the difficulties in performing RT-PCR test in all cases related to the absence of swaps and reagents, the different protocols between regions in case of suspected infection in asymptomatic patients, and the overbooking of some health facilities or similar during the pandemic. However, the higher incidence of interstitial pneumonia in the COVID period and the majority of patients who performed RT-PCR that resulted positive for COVID-19 infection may be considered indirect signs suspicious of COVID infection.

Moreover, several studies showed the high accuracy of chest CT in comparison with RT-PCR in the diagnosis of COVID-19 [8, 31] and the low sensitivity of RT-PCR.

Other limitations of this work were the retrospective design of the study, the heterogeneity in patient enrollment at the national level with geographic differences (high adhesion in northern centers), and the arbitrary choice of different intervals of time of the three periods analyzed also due to organization reasons.

In conclusion, with this study, we have demonstrated that during the COVID-19 pandemic, a statistically significant increase of interstitial pneumonia at [^18^F]FDG PET/CT compared with a pre-COVID period and a control COVID-19-free period was registered in a country with high prevalence of infection. These findings were directly associated with the geographic virus diffusion with high rate in northern regions. In most cases, interstitial pneumonia suspicious for COVID-19 was FDG-avid. This is the first study that represents a national experience in a country with high prevalence of COVID-19 infection.

## Supplementary information

ESM 1 (JPG 137 kb)
