# Supplementary figures and images for: Prevalence of interstitial pneumonia suggestive of COVID-19 at 18F-FDG PET/CT in oncological asymptomatic patients in a high prevalence country during pandemic period: a national multi-centric retrospective study
Source: Eur J Nucl Med Mol Imaging. 2021 Feb 9;48(9):2871–82. doi: 10.1007/s00259-021-05219-0 (PMC7871520; doi:10.1007/s00259-021-05219-0)

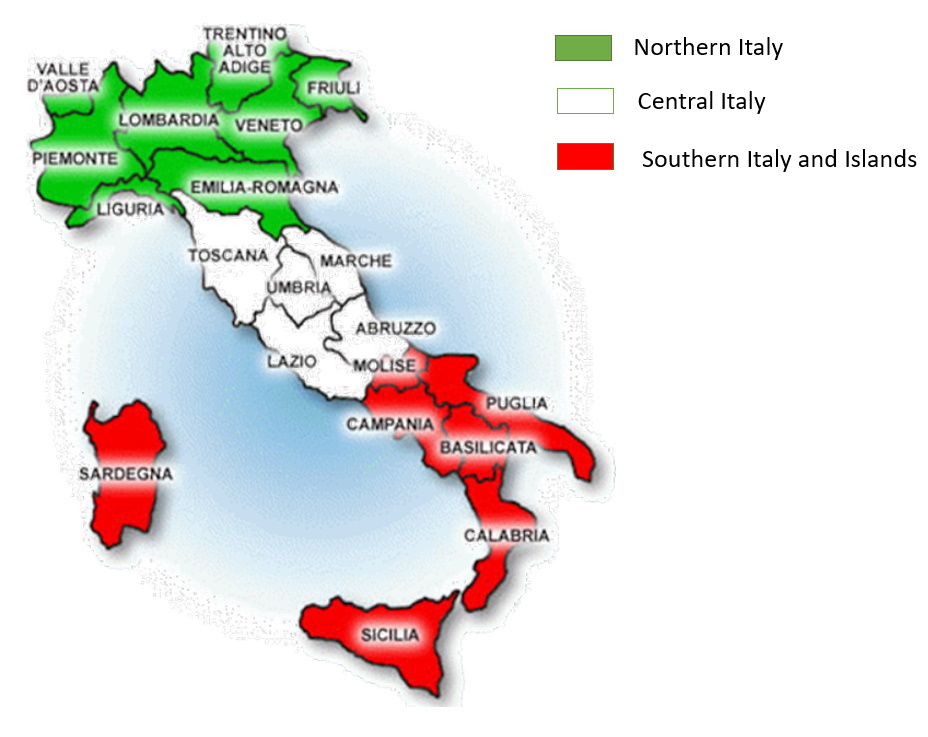

Supplement: Supplementary file 1 — (JPG 137 kb) [file 259_2021_5219_MOESM1_ESM.jpg]
